# Gravitational change-induced alteration of the vestibular function and gene expression in the vestibular ganglion of mice

**DOI:** 10.1186/s12576-024-00939-y

**Published:** 2024-09-18

**Authors:** Murat Bazek, Motoya Sawa, Kazuhiro Horii, Naotoshi Nakamura, Shingo Iwami, Chia-Hsien Wu, Tsuyoshi Inoue, Fumiaki Nin, Chikara Abe

**Affiliations:** 1 Department of Physiology, Gifu University Graduate School of Medicine, 1-1 Yanagido, Gifu, 501-1194 Japan; 2 Interdisciplinary Biology Laboratory (iBLab), Division of Natural Science, Graduate School of Science, Nagoya University, Aichi, Japan; 3 Department of Physiology of Visceral Function and Body Fluid, Nagasaki University Graduate School of Biomedical Sciences, Nagasaki, Japan

**Keywords:** Hypergravity, Plasticity, RNA-seq, Vestibular system, Labyrinth

## Abstract

Gravity has profoundly influenced life on Earth, yet how organisms adapt to changes in gravity remains largely unknown. This study examines vestibular plasticity, specifically how the vestibular system responds to altered gravity. We subjected male C57BL/6J mice to hypergravity (2 G) followed by normal gravity (1 G) to analyze changes in vestibular function and gene expression. Mice showed significant vestibular dysfunction, assessed by righting reflex tests, which persisted for days but reversed at 1 G after exposure to 2 G. Gene expression analysis in the vestibular ganglion identified significant changes in 212 genes out of 49,585 due to gravitational changes. Specifically, 25 genes were upregulated under 2 G and recovered at 1 G after 2 G exposure, while one gene showed the opposite trend. Key neural function genes like Shisa3, Slc25a37, Ntn4, and Snca were involved. Our results reveal that hypergravity-induced vestibular dysfunction is reversible and highlight genes critical for adaptation.

**Supplementary Information:**

The online version contains supplementary material available at 10.1186/s12576-024-00939-y.

## Introduction

Gravity, a constant environmental factor on Earth since the origin of life, has shaped the course of life's evolution for adaptation. Despite the difficulty of manipulating gravity, many aspects of this adaptation remain poorly understood. As humanity ventures towards lunar and Martian exploration, comprehending how various organisms, including ourselves, adapt to novel gravitational environments becomes even more essential.

The vestibular system serves as a critical organ for detecting gravitational changes in various organisms on Earth, including fish, reptiles, birds, and mammals. This system comprises two main types of peripheral sensors located in the inner ear: the semicircular canals and the otolith organs [1]. The semicircular canals detect angular accelerations, while the otolith organs are responsible for detecting linear accelerations. Signals from each sensor are transmitted to the brain, specifically to the vestibular nuclear complex, via the vestibular nerve.

The vestibular system exhibits a remarkable feature: plasticity. Animal studies have demonstrated that the microgravity environment alters surface righting and swimming behavior in a time-dependent manner [2, 3]. Additionally, structural synaptic plasticity has been detected in the otolith organs, including the utricle, in mice after spaceflight [4]. Furthermore, we have previously reported that hypergravity environments induce vestibular plasticity in rats, altering the function of the autonomic response and motor coordination mediated by the vestibular system [5–9]. Interestingly, these functional changes induced by exposure to hypergravity environments are reversible upon returning to a 1 G environment [7]. Although a similar reversible effect is observed in astronauts as same as rodents, the molecular mechanisms underlying this physiological response remain elusive. Accordingly, the present study investigates the changes in physiological and molecular responses induced by gravitational changes, including exposure to 2 G and unloading (2 G exposure followed by 1 G).

## Methods

### Animals

Male C57BL/6J mice (8 weeks old) were purchased from Japan SLC (*n* = 45) and housed with same-sex littermates in a temperature-controlled environment (22–24 °C) with ad libitum access to food and water. All procedures were conducted according to the "Guiding Principles for Care and Use of Animals in the Field of Physiological Science" set by the Japan Physiological Society and approved by the Animal Research Committee of Gifu University (AG-P-N-20240065).

### Anesthesia and postoperative management

Surgery was performed under aseptic conditions. Mice were anesthetized intraperitoneally with ketamine (120 mg/kg) and xylazine (12 mg/kg). Anesthesia depth was confirmed by the absence of pedal withdrawal reflex. Additional anesthetic (10% of the original dose) was administered intraperitoneally as needed. Body temperature was maintained at 37.0 ± 0.5 °C with a servo-controlled temperature pad. After surgery, mice received postoperative injections of atipamezole (an α2-adrenergic antagonist, 2 mg/kg, s.c.), penicillin G potassium (3000 U/kg, s.c.), and ketoprofen (4 mg/kg, s.c.). Mice were subsequently housed in groups of four per cage under a 12:12 h light–dark cycle. The room temperature was maintained at 24 ± 1 °C.

### Vestibular lesion

The vestibular lesion (VL) was performed using the previously described method [10]. Following aseptic surgical procedures, the tympanic membrane, malleus, incus, and stapes were carefully removed. Labyrinthine fluid was aspirated, and PERIODON (Neo Dental International, Japan) was placed through the oval window. This medication containing dibucaine and paraformaldehyde induces protein denaturation in the otolith organs, leading to vestibular lesions. Sham-operated mice underwent similar procedures, except the auditory ossicles remained intact. We allowed 21 days for recovery after the surgery, and the success of VL was evaluated using a swimming test. Mice were unable to swim if VL was successfully achieved.

### Exposure to hypergravity

We used the previously described method to create hypergravity (2 G) [10]. Mice were exposed to 2 G using a gondola-type rotating box with a 50 cm arm (Takei Scientific Instruments Co., Ltd., Japan). Centrifugation at 55 rpm was used to achieve 2 G. Mice were housed (*n* = 6 in a cage) under a 12:12 h light–dark cycle with ad libitum access to food and water. Room temperature was maintained at 24 ± 1 °C.

### Physiological test of vestibular function

The physiological assessment was conducted using the righting test, following the previously described method [5, 11]. A mouse was dropped from 50 cm above the ground. The mouse starts falling upside-down (the dorsal side of the mouse toward the ground). We measured the distance to which the mouse returned to its original position (ventral side of the mouse toward the ground) using a high-speed camera (iPhone, Apple). If vestibular function worsened, the time required was longer, or the righting reflex was not observed. The trial was repeated five times for each mouse, and data were averaged except for the largest and smallest values.

### Experiment design

**Fig. 1:**
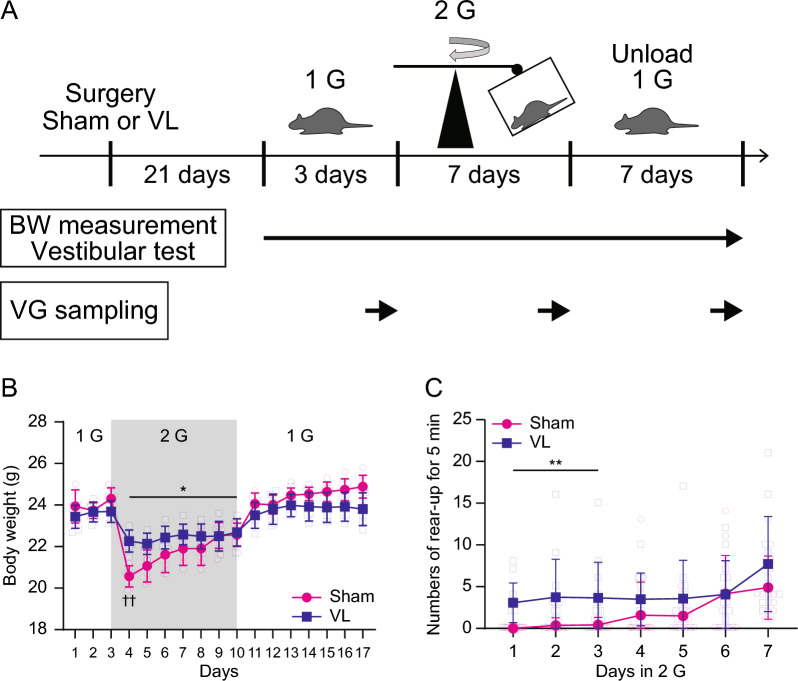
Experimental design and changes in body weight and activity in a 2 G environment. **A** Timeline of the experiment. BT denotes body temperature, BW denotes body weight and VG denotes vestibular ganglion. **B** The effect of hypergravity load and unloading on changes in body weight in Sham (*n* = 6) and VL (*n* = 6) mice. Statistics: two-way ANOVA with Dunnett’s comparisons test or Tukey’s multiple comparisons test. The symbols “*” indicates comparisons vs. Day 3, and “†” indicates comparisons between Sham and VL. Single or double significant symbols indicate *P* < 0.05 and *P* < 0.01, respectively. **C** The number of rear-ups for 5 min from the start of each 2 G load in Sham (*n* = 12) and VL (*n* = 12) mice. Statistics: two-way ANOVA with Tukey’s multiple comparisons test. The symbol “*” indicates comparisons between Sham and VL. A double significant symbol indicates *P* < 0.01

Mice were housed in a 1 G environment for 3 days, followed by exposure to the 2 G environment for 7 days, and then returned to the 1 G environment for another 7 days (Fig. 1A). Body weight was measured daily in Sham (*n* = 6) and VL mice (*n* = 6). Righting reflex function was measured daily in 1 G (*n* = 6), 2 G (*n* = 6), Unload (*n* = 6) and VL (*n* = 6) mice during cage changes with measurement of body weight. Cage changes and measurements required approximately 30 min in 1 G environment.

### RNA purification and quantification of the vestibular ganglion

Mice were divided into three groups (*n* = 3 per group): 1 G, 2 G, and Unload. The Unload group consisted of mice housed in a 1 G environment for 7 days following exposure to the 2 G environment for 7 days. All mice underwent isoflurane anesthesia, followed by bilateral vestibular ganglion sampling in the labyrinth. RNA was purified using RNeasy FFPE Kit (73504, Qiagen, Hilden, Germany) with modifications in procedures. The samples were dissolved in 40 μL of Buffer PKD and 3 μL of Proteinase K solution was added then incubated at 56 °C for 15 min, followed by incubation at 80 °C for 10 min. Reverse-crosslinked RNA samples were combined with 120 μL TRIzol LS Reagent (1029028, Ambion, Life Technologies, Thermo Fisher Scientific, Carlsbad, CA, USA) then 32 μL of chloroform (035-02616, Wako, Osaka, Japan) was added. The samples were vortex-mixed and the aqueous phase was separated by centrifugation at 13,000 rpm for 15 min at 4 °C. Supernatant was transferred to 1.5 mL microfuge tubes with 10 μg glycogen (G1767, Sigma–Aldrich, St. Louis, MO, USA) and 80 μL of 2-propanol (168-21675, Wako, Osaka, Japan) was added to precipitate RNA. The pellet was recovered by centrifugation at 13,000 rpm for 15 min at 4 °C and rinsed twice with 80% ethanol as described in the user manual. The final pellet was resuspended in 6 μL of water.

### RNA-sequencing library preparation and sequencing

The quality of purified RNA was examined by RNA 6000 Pico Kit (5067-1513, Agilent Technologies, Waldbronn, Germany). Purified RNA (3.5 μL) was converted into the sequencing library using SMARTer Stranded Total RNA-Seq Kit Pico Input Mammalian (635005, Takara Bio, Mountain View, CA, USA), at half-volume scale following the user manual. The template amount was verified before the second PCR step by taking a 1 μL aliquot of library reaction and performing real-time PCR with Fast SYBR Green Master Mix (4385612, Applied Biosystems, Thermo Fisher Scientific, Foster City, CA, USA) to adjust the PCR template amount and PCR cycle number in library amplification. The final PCR step was performed with index primers. PCR products were purified and eluted in 16 μL of Stranded Elution Buffer combined with water at a 1:1 ratio.

Library preparation to obtain RNA profiles of samples was performed using the NEBNext rRNA Depletion Kit (Human/Mouse/Rat) and the NEBNext Ultra Directional RNA Library Prep Kit for Illumina (E6310 and E7420, New England Biolabs, Ipswich, MA, USA). Final PCR products were purified and eluted in EB buffer from the QIAquick PCR Purification Kit (28106, Qiagen, Hilden, Germany).

Library quality and concentration were determined using the High Sensitivity DNA Kit (5067-4626, Agilent Technologies, Waldbronn, Germany). Libraries were mixed at equal molar concentrations for multiplex sequencing. Sequencing was performed with the NextSeq500 System (Illumina, San Diego, CA, USA), using the High Output Kit v2 (75 Cycles, FC-404-2005) to obtain 36-base paired-end reads at the Tsukuba i-Laboratory (Tsukuba, Japan). FASTQ files were generated by BaseSpace Onsite.

### Sequencing data analysis

Reads in FASTQ files were imported to CLC main Workbench (CLC-MW, ver.24.0.1, Qiagen, Germantown, MD, USA), mapped to the mouse reference genome (mm10), and quantified using a 49,585-gene annotation set (downloaded from the CLC-MW server) to obtain the Total Count and Reads Per Kilobase of transcript per Million mapped reads (RPKM) values. For the analysis of samples, RPKM values were normalized by the quantile method using CLC-MW, and difference and fold change values were calculated. For the heatmap, normalized RPKM values were log-2 transformed after adding a pseudocount of 0.01. Total count values were normalized by the quantile method and analyzed by Gaussian Statistical Analysis with the ANOVA option in CLC-MW. For the heatmap and scatter plot, normalized total count values were log-2 transformed after adding a pseudocount of 1.

### Heatmap and gene clustering

Heatmaps were created using Morpheus (https://software.broadinstitute.org/morpheus/). Normalized Log2 total count values for filtered genes were exported from CLC-MW and imported to Morpheus. Clustering trees were produced by Euclidean complete linkage.

### Pathway and gene ontology analysis

Pathway analysis was performed using Metascape (http://metascape.org/gp/index.html) with the Expression Analysis mode. Gene Ontology analysis was performed using Reactome (https://reactome.org/). Both tools were used with default parameter settings.

### Statistical analysis

All datasets were checked for normality using either the D’Agostino-Pearson omnibus normality or Kolmogorov–Smirnov test. Equal variances were successively examined using the Brown–Forsythe test. The normality and equal variance were satisfied in all cases, therefore statistical significance was evaluated using the two-way ANOVA. Tukey’s or Bonferroni’s and Dunnett’s multiple comparison tests were applied as necessary. When Dunnett’s multiple comparison test was applied, the pre-loading value (Day 3) was used as a reference value to account for acclimation to the experiment, even in the 1 G environment. All values are expressed as the mean ± the standard error of the mean, and statistical significance was set at *P* < 0.05.

## Results

Hypergravity loading (2 G) resulted in decreased body mass in both Sham and VL mice (Fig. 1B). This decrease was significant compared to pre-loading (Day 3) values and persisted throughout the 2 G exposure. Body mass returned to baseline levels when the mice were returned to a 1 G environment. Interestingly, only the first day of 2 G loading showed a significant difference in body mass between Sham and VL mice. This difference disappeared from the second day onward.

The number of rear-up events was counted during the first 5 min of 2 G loading in both Sham and VL mice (Fig. 1C). A significant difference in numbers of rear-up was observed until the third day of loading. Thereafter, the difference between the groups disappeared.

**Fig. 2:**
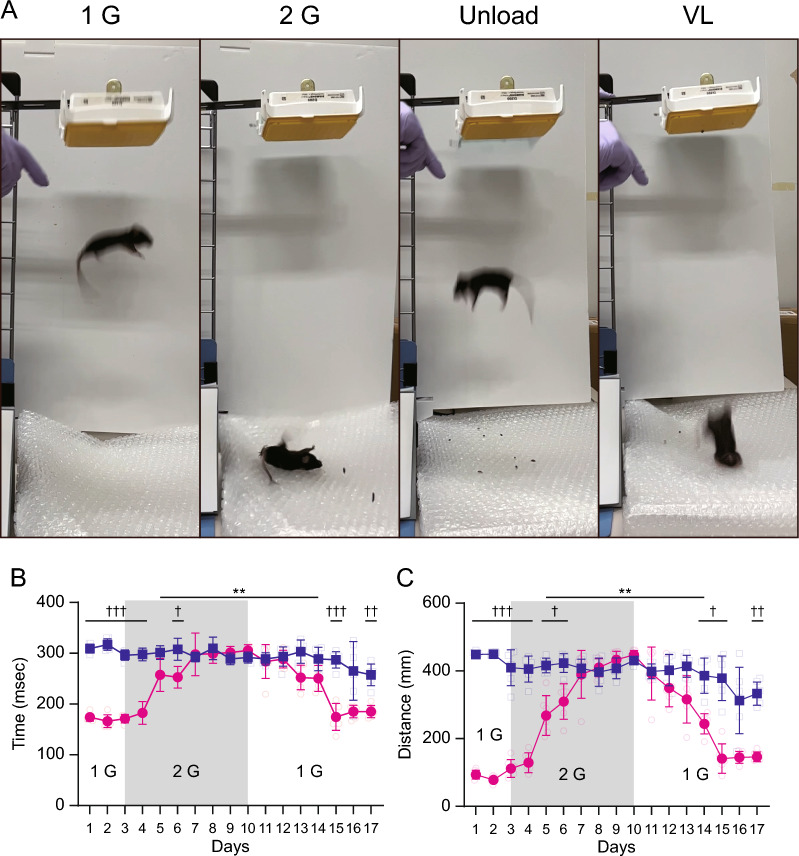
Changes in the vestibular function. **A** Changes in skills of righting reflex by hypergravity change. Representative pictures of the righting reflex in 1 G, 2 G, unload and VL mice. **B**, **C** Summarized data of righting reflex in 1 G (*n* = 6), 2 G (*n* = 6), unload (*n* = 6) and VL (*n* = 6) mice. The value indicates the time (**B**) and distance (**C**) which the mouse requires to revert from the dropped posture to the normal postural. Statistics: two-way ANOVA with Dunnett’s comparisons test or Tukey’s multiple comparisons test. The symbols “*” indicates comparisons vs. Day 3, and “†” indicates comparisons between Sham and VL. Single, double or triple significant symbols indicate *P* < 0.05, *P* < 0.01 and *P* < 0.001, respectively

The vestibular function was assessed using the righting reflex test in both Sham and VL mice (Fig. 2). The time and distance required for the mice to return to a normal position in the righting reflex test gradually worsened following exposure to a 2 G environment. Significant deterioration was observed 2 days after exposure to the 2 G environment, and function in Sham mice became similar to that in VL mice 4 days after exposure. The impairment of the vestibular system persisted for 4 days after unloading.

**Fig. 3:**
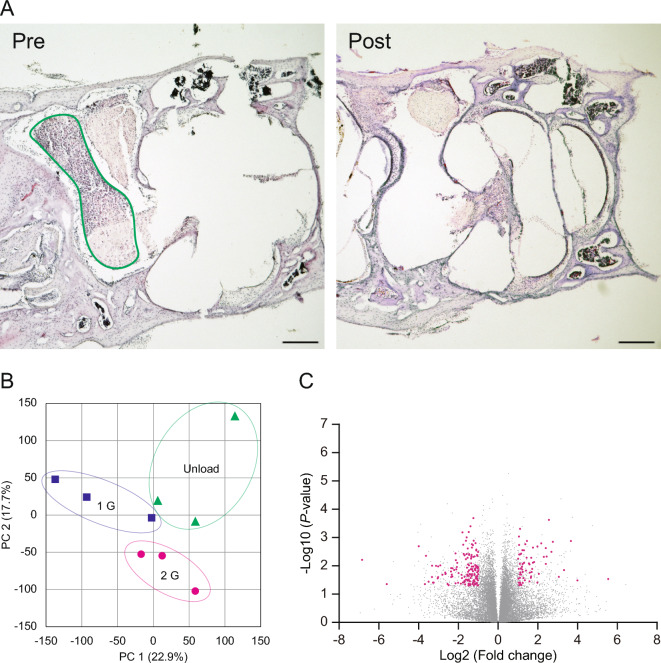
Sampling of the vestibular ganglion. **A** Hematoxylin–Eosin staining of a section of bony labyrinth. The area with green colored border is the vestibular ganglion. The vestibular ganglion which is for RNA-seq was correctly sampled. A scale bar indicates 500 μm. **B** PCA plot of DEGs. **C** Volcano plot which was obtained by ANOVA among 3 groups including 1 G, 2 G and Unload using max fold change. The plots with magenta were significantly changed more than twofold

**Fig. 4:**
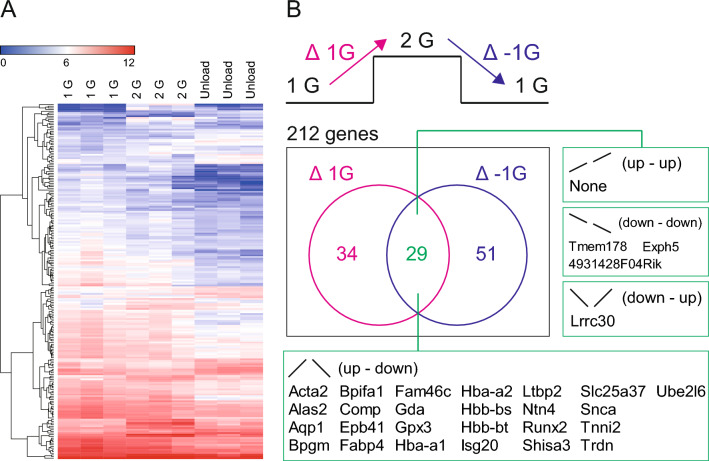
Changes in gene expression in the vestibular ganglion. **A** Heatmap representation of RNA expression in the vestibular ganglion. **B** Venn diagram comparing altered 114 genes, which were changed by increase or decrease in gravity. The common altered genes by gravitational change were 29

To explore alterations in gene expression within the vestibular ganglion, tissue samples were collected from the labyrinth. The accuracy of tissue sampling was confirmed through histological examination (Fig. 3A). As depicted in the PCA plot of Fig. 3B, distinct gene expression profiles were observed within the vestibular ganglion across the 1 G, 2 G, and Unload groups. A total of 49,585 genes were examined (Table S1), with a focus on those exhibiting significant differences (Fig. 3C). Among these, 212 genes met the predefined criteria (ANOVA fold change > 2, ANOVA difference > 20, and *P* < 0.05, Table S2). The comprehensive changes in gene expression, encompassing both upregulation and downregulation, are summarized in the heatmap (Fig. 4A). Statistical analysis revealed that 63 out of 49,585 genes were significantly changed in response to increased gravity (Δ 1 G), while 80 genes were significantly changed across groups in response to decreased gravity (Δ −1 G). Notably, the observed alterations in gene expression paralleled changes in the righting reflex, with 25 out of 49,585 genes showing specific significant upregulated at 2 G, and these reverted upon unloading (Fig. 4B). One gene exhibited significant downregulated in response to 2 G loading, and its expression level reverted upon unloading (Fig. 4B).

## Discussion

The major findings of this study are as follows: (1) one day unloading was required recovery from decrease in body weight induced by 2 G exposure, (2) five days unloading was required recovery from decrease in vestibular function induced by 2 G exposure, (3) 212 specific genes were significantly upregulated or downregulated by changes in gravity and (4) 29 common genes were changed by gravitational alteration.

Previous studies have established that hypergravity load can lead to a reduction in body weight in rats [12] and mice [5]. In the present study, a significant difference in body weight was observed after just one day of exposure to 2 G conditions between Sham and VL mice. Body weight is determined by a balance between food intake and energy expenditure. Our prior research indicated that food intake decreased significantly under 2 G exposure in Sham mice but not in VL mice [13]. Changes in gravitational force, whether hypergravity or microgravity, can induce symptoms such as motion sickness, implicating the stimulation to the vestibular system [14]. Notably, VL animals did not exhibit hypergravity-induced motion sickness [15], and chemogenetic inhibition of the central vestibular system in mice eliminated the decrease in food intake induced by rotational motion [16]. This suggests that chronic stimulation of the peripheral vestibular apparatus may contribute to the decrease in body weight observed under 2 G exposure in Sham mice. However, it is worth noting that a significant decrease in body weight was also observed in VL mice. It is suggested that the decrease in body weight might be attributed to an increase in energy expenditure because there is no change in food intake in VL mice [13].

In contrast to the changes in body weight during 2 G exposure, vestibular function significantly deteriorated two days after exposure in Sham mice. However, there was no discernible difference in vestibular function between Sham and VL mice three days after 2 G exposure, a finding consistent with the equalization of rearing behavior in the 2 G environment (Fig. 1C). This suggests that the ability to detect gravitational information was impaired by 2 G exposure in Sham mice, rendering them unable to appropriately perceive gravitational cues under 1 G conditions. Notably, the deteriorated vestibular function was restored by unloading in the present study. Prior research from our laboratory demonstrated that vestibular function, as assessed by rotarod performance, was restored after 2 weeks of exposure to 2 G followed by 1 week of unloading in rats [7]. Consistent with these observations, our current study found that 5 days of unloading were necessary to restore vestibular function following just 1 week of 2 G exposure, despite the fact that Sham mice were bred and raised under 1 G conditions. Interestingly, while body weight was restored after just 1 day of unloading, indicating a different recovery timeline for vestibular-related metabolism compared to motor function.

In this study, we investigated alterations in RNA expression within the vestibular ganglion across mice subjected to 1 G, 2 G, and unload conditions. Statistical analysis revealed significant changes in 212 out of 49,585 genes in response to gravitational loading or unloading. Our findings demonstrated that vestibular function deteriorated under 2 G loading but was restored upon unloading. Consequently, we identified 25 genes that were upregulated by 2 G and subsequently downregulated by unloading, along with one gene that exhibited the opposite pattern. These 26 genes showed corresponding changes to alterations in vestibular function induced by gravitational variations. Among these genes, four, namely Shisa3, Slc25a37, Ntn4 (Netrin G1), and Snca, are known to be associated with the neural system in mice. Shisa3, for instance, is a protein involved in the development and function of synapses between neurons [17]. Similarly, Slc25a37 functions as a transporter protein regulating glutamate, a primary excitatory neurotransmitter in the brain [18].

Netrins comprise a family of proteins crucial for axon guidance during development and also contribute to adult neurogenesis and synaptic plasticity, both pivotal for neural system function [19]. Ntn4, specifically, is implicated in synaptic plasticity, influencing the structural and functional dynamics of synapses and, consequently, their signaling efficiency. The Snca gene in mice plays a multifaceted role in neural plasticity, governing the brain's adaptability and changeability [20]. Encoded by Snca, alpha-synuclein is believed to regulate synaptic function and plasticity in healthy brains, with some studies suggesting its involvement in maintaining proper synaptic structure and function. Its high concentration in presynaptic terminals suggests a role in regulating the pool of synaptic vesicles, which store neurotransmitters for transmission. Given that the vestibular ganglion comprises a cluster of cell bodies within the vestibular nerve, plastic alterations in the vestibular system likely occur at the synapse, projecting to the vestibular nucleus complex. These gene expression changes shed light on the molecular mechanisms underlying vestibular function modulation in response to gravitational shifts.

On the other hand, it might be speculated that the increase in Snca expression in the vestibular ganglion contributes to the decrease in body weight during 2 G exposure. Snca is implicated in neurodegenerative diseases like Parkinson's disease, where patients often experience nausea and vomiting [21]. Previously, we demonstrated that exposure to hypergravity induces motion sickness via the vestibular system [15]. Additionally, food intake was significantly suppressed in mice during 2 G exposure in a previous study [5]. Therefore, if Snca in the vestibular ganglion is involved in motion sickness and the emetic response, the reduction in food intake due to these symptoms may contribute to the decrease in body weight during 2 G exposure.

In the present study, 143 genes exhibited changes in expression in unloading conditions compared with 1 G mice, with 24 genes upregulated and 119 genes downregulated. Notably, 23 of the 24 upregulated genes and 95 of the 119 downregulated genes did not change with 2 G exposure. This suggests that these genes might play a role in indirect functional compensation. Specifically, to recover from vestibular dysfunction induced by 2 G exposure, genes that were unaffected by 2 G may be recruited for functional compensation. It is known that non-vestibular inputs, including visual, muscular, tactile, and intestinal systems, are recruited to compensate for vestibular inflammation-induced dysfunction [22, 23]. This compensation also occurs for the gravitational change-induced vestibular dysfunction [6]. Although this is speculative, the observed gene expression changes might be crucial for interaction with the non-vestibular system during compensation. Future studies should investigate this possibility.

## Supplementary Information

Additional file 1.

Additional file 2.

## Data Availability

The data underlying this article will be shared on reasonable request to the corresponding author.
